# Clinical and radiological aspects of bilateral temporal abnormalities: pictorial essay

**DOI:** 10.1590/0100-3984.2019.0134

**Published:** 2021

**Authors:** Heloisa Sisconeto Bisinotto, Vinicius Menezes Jarry, Fabiano Reis

**Affiliations:** 1 Universidade Estadual de Campinas (Unicamp), Campinas, SP, Brazil.

**Keywords:** Temporal lobe/diagnostic imaging, Computed tomography, Magnetic resonance imaging, Lobo temporal/diagnóstico por imagem, Tomografia computadorizada, Ressonância magnética

## Abstract

The temporal lobes are vulnerable to several diseases, including infectious, immune-mediated, degenerative, vascular, metabolic, and neoplastic processes. Therefore, lesions in the temporal lobes can pose a diagnostic challenge for the radiologist. The temporal lobes are connected by structures such as the anterior commissure, corpus callosum, and hippocampal commissure. That interconnectedness favors bilateral involvement in various clinical contexts. This pictorial essay is based on a retrospective analysis of case files from a tertiary university hospital and aims to illustrate some of the conditions that simultaneously affect the temporal lobes, as well as to define some neuroimaging elements that may be useful for the differential diagnosis of these diseases. Using computed tomography and magnetic resonance imaging scans, we illustrate the neuroradiological findings in confirmed cases of human herpesvirus 1, central nervous system tuberculosis, autoimmune encephalitis, Alzheimer's disease, frontotemporal dementia, mesial temporal sclerosis, stroke, kernicterus, megalencephalic leukoencephalopathy with subcortical cysts, low-grade glioma, and secondary lymphoma, the objective being to emphasize the importance of these imaging methods for making the differential diagnosis.

## Abstract (pt)

Os lobos temporais são vulneráveis a diversas doenças, incluindo processos infecciosos, imunomediados, degenerativos, vasculares, metabólicos e neoplásicos, de modo que lesões nessa região podem representar um desafio diagnóstico ao radiologista. Estruturas como a comissura anterior, o corpo caloso e a comissura hipocampal são responsáveis por interligarem os lobos temporais, favorecendo o acometimento bilateral em diferentes contextos clínicos. Este artigo utilizou-se da análise retrospectiva de casos do arquivo didático de um hospital universitário terciário e tem como objetivo ilustrar algumas das condições que acometem simultaneamente os lobos temporais, bem como definir alguns elementos de neuroimagem que podem ser úteis para o diagnóstico diferencial dessas doenças. Ilustramos os achados neurorradiológicos de tomografia computadorizada e ressonância magnética de casos com diagnóstico confirmado de encefalite por herpes vírus humano tipo 1, neurotuberculose, encefalite autoimune, doença de Alzheimer, demência frontotemporal, esclerose temporal mesial bilateral, acidente vascular cerebral, kernicterus, leucoencefalopatia megalencefálica com cistos subcorticais, glioma de baixo grau e linfoma secundário, a fim de enfatizar a importância dos métodos de imagem para o diagnóstico diferencial.

## INTRODUCTION

**Figure 1:**
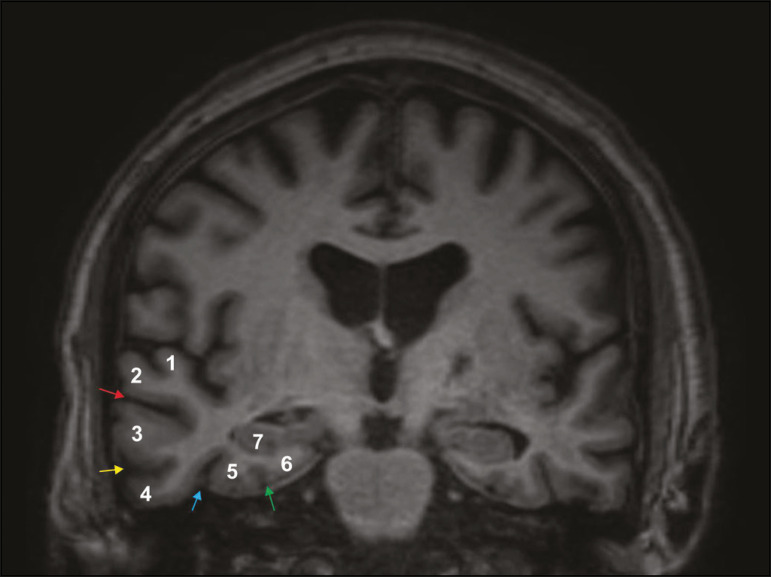
Coronal T1-weighted MRI sequence showing the normal anatomy of the temporal lobe. 1, transverse temporal gyrus; 2, superior temporal gyrus; 3, middle temporal gyrus; 4, inferior temporal gyrus; 5, lateral occipitotemporal gyrus; 6, medial occipitotemporal/parahippocampal gyrus; 7, hippocampus. Red arrow, superior temporal sulcus; yellow arrow, inferior temporal sulcus; blue arrow, occipitotemporal sulcus; green arrow, collateral sulcus.

The temporal lobe can be divided into the neocortex and the mesial temporal lobe. The neocortex corresponds to the lateral inferior surface of the lobe and is related to sight, hearing, and speech processes. The mesial temporal lobe, situated medially, is part of the limbic system; it plays a role in the control of emotions, behavior, and memory, as well as regulating neuroendocrine and autonomic functions (Figure 1). Structures such as the anterior commissure, corpus callosum, and hippocampal commissure are responsible for interconnecting the temporal lobes, favoring bilateral involvement in various clinical contexts^(1)^.

Patients with lesions in the temporal lobe can present mental confusion or a reduced level of consciousness; imaging examinations are essential for their appropriate evaluation and early etiological diagnosis. The use of advanced magnetic resonance imaging (MRI) sequences such as hydrogen proton spectroscopy and perfusion MRI contributes to the differential diagnosis of such lesions, as shown in Table 1 ^(2-12)^. Some of the diseases that can affect the temporal lobes are detailed below, with an emphasis on neuroradiological findings to highlight the importance of neuroimaging in the differential diagnosis.

**Table 1:** Summary of diseases with bilateral involvement of the temporal lobes.

Disease	Main anatomical changes	Accompanying changes	Perfusion	Spectroscopy
Herpes simplex encephalitis	Bilateral, asymmetric T2/FLAIR hyperintensity in the temporal lobes	Cortical hemorrhage, gyriform enhancement, and restricted diffusion on DWI	Hypoperfusion	Reduced NAA/Cr ratio and increased Cho/Cr ratio. There can be Lip and Lac**^(2,3)^**
Neurotuberculosis	Enhancement of leptomeninges and dura mater, tuberculomas	Hydrocephalus, ventriculitis, vasculitis, infarction, venous thrombosis, neuropathies	Variable	Lip and Lac peak**^(4,5)^**
Limbic encephalitis	Bilateral, asymmetric T2/FLAIR hyperintensity in mesial temporal structures	Involvement of basal ganglia, enhancement, and restricted diffusion on DWI	Hypoperfusion	Reduced NAA. Increased Cho, Lac, and MI**^(6)^**
Alzheimer's disease	Reduction in the volume of the mesial temporal structures (especially the hippocampus) disproportional to the atrophy in the remaining parenchyma	Atrophy of the superior parietal lobule	Hypoperfusion	Reduced NAA and NAA/Cr ratio in the cingulate gyrus and hippocampi. Increased MI/Cr ratio in the cingulate gyrus and parietal cortex**^(7)^**
Frontotemporal dementia	Selective atrophy of the frontal or tem­poral lobes	Atrophy predominantly on the left in primary progressive aphasia	Hypoperfusion	Reduced NAA/Cr ratio and increased MI/ Cr ratio in the frontal cortex**^(7)^**
Mesial temporal sclerosis	T2/FLAIR hyperintensity in the hippocampus with loss of volume and dilatation of the temporal horn (bilateral in 10% of cases)	Atrophy of the amygdala, fornix, mammillary body and entorhinal cortex. Loss of cortical/subcortical differentiation in the temporal pole	Hypoperfusion	Reduced NAA in the affected temporal lobe and in the hippocampus**^(8)^**
Cerebrovascular disease	Loss of cortical/subcortical differentiation in the involved vascular territory. T2/FLAIR hyperintensity. Early restrict­ed diffusion on DWI	Hyperdense artery sign, gyriform enhancement	Hypoperfusion	In the acute phase, there is increased Cho, Lip, and Lac, with reduced NAA**^(5,9)^**
Kernicterus	T2/FLAIR hyperintensity with hippocampal atrophy in the chronic phase	Change in the signal of the globus pallidus and subthalamic nuclei	Hypoperfusion	Increased Tau, Glx, and MI, with reduced Cho**^(10)^**
Megalencephalic leukoencephalopathy with subcortical cysts	Diffuse hyperintense signal of deep white matter sparing basal ganglia and cerebellum	Subcortical cysts evident initially in the temporal lobes and later in the frontal and parietal lobes	Hypoperfusion	Increased Cho and MI, with reduced NAA, in the early stage. Increased Lac in the advanced stage**^(11)^**
Lowgrade diffuse astrocytoma	T1 hypointensity and T2/FLAIR hyperintensity, infiltration of the cortex, no enhancement, and no restricted diffu­sion on DWI	No evidence of necrosis or hemorrhagic components	Hypoperfusion (rCBV < 1.75). Hyperperfusion if anaplastic/ high grade	Increased MI, slightly increased Cho (Cho/Cr ratio < 2), reduced NAA, and ab­sence of Lac**^(3)^**
Lymphoma	T2/FLAIR hypointensity with restricted diffusion on DWI. Intense, homogeneous enhancement	In immunocompetent patients, a necrotic component is rarely seen	Hypoperfusion	Increased Cho, Lip, and Lac. Reduced NAA**^(12)^**

rCBV, relative cerebral blood volume; NAA, N-acetylaspartate; Cr, creatine; Cho, choline; Lip, lipids; Lac, lactate; MI, myo-inositol; Tau, taurine; Glx, glutamate and glutamine.

## INFECTIOUS ETIOLOGY

Acute infectious encephalitis is a serious condition that can be caused by numerous pathogens, herpes simplex encephalitis being the most common. Human herpesvirus 2 and cytomegalovirus are classically associated with neonatal encephalitis. Most cases that occur after the neonatal period are due to reactivation of human herpesvirus 1^(13)^. In immunocompromised patients, human herpesvirus 8 can have a presentation similar to that of human herpesvirus 1.

**Figure 2:**
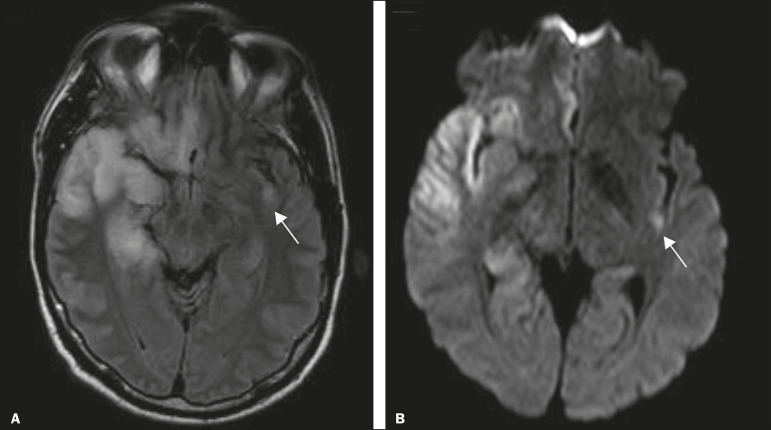
A 46-year-old male patient with headache, fever, and mental confusion. A: Axial FLAIR MRI sequence showing a hyperintense signal in both temporal lobes (arrow), more pronounced on the right. B: DWI with bilateral restricted diffusion (arrow). Positivity for human herpesvirus 1 on polymerase chain reaction of the cerebrospinal fluid.

Cranial computed tomography (CT) has low sensitivity in the initial stages of encephalitis. However, when alterations are visible on cranial CT, they are associated with serious cerebral damage and a worse prognosis. The imaging method of choice is MRI. On T2 and fluid attenuated inversion recovery (FLAIR) weighted images (WI), cortical and subcortical temporal lobe hyperintensity is a finding characteristic of herpes simplex encephalitis. Encephalitis can initially be unilateral, thereafter evolving to asymmetric bilateral involvement (Figure 2). Isolated involvement of the hippocampus is not a common finding; it should raise the suspicion of differential diagnoses such as limbic encephalitis and status epilepticus. On MRI, foci of cortical bleeding, areas of restricted diffusion-on diffusion-weighted imaging (DWI)-and gyriform contrast enhancement can also be observed^(1,13,14)^.

**Figure 3:**
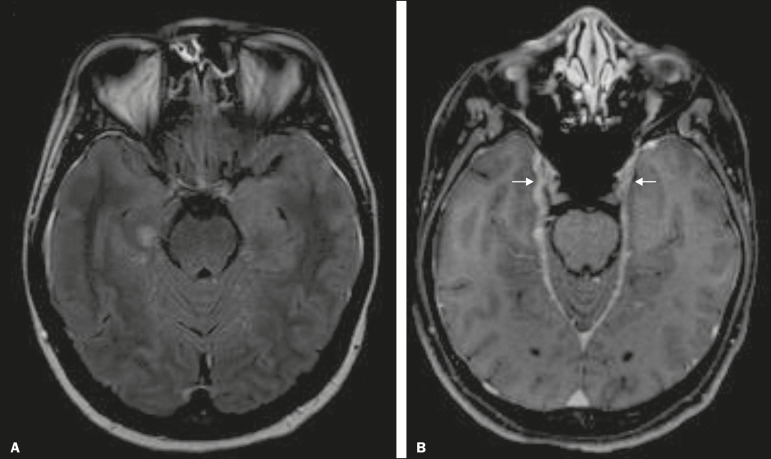
A 35-year-old, HIV-positive female patient who presented with a one-week history of fever and left frontoparietal headache. Cerebrospinal fluid analysis showed high protein levels, lymphocytic leukocytosis, reduced glucose, and increased adenosine deaminase, favoring a diagnosis of neurotuberculosis. Axial FLAIR-weighted and contrast-enhanced axial T1-weighted MRI sequences (A and B, respectively) showing thickening of the dura mater (arrows), which was more pronounced in the right temporal region, where the FLAIR-weighted shows a change in the signal. The patient showed symptom improvement, the follow-up examinations performed one year after the onset of symptoms showing resolution of the radiological aspects (images not shown).

By hematogenous spread, tuberculosis can also involve the central nervous system (CNS), that form being seen in up to 5% of patients with tuberculosis, although the incidence is higher among those who are immunocompromised. Although tuberculous leptomeningitis is the most common presentation, CNS tuberculosis can also involve the meninges as a whole, resulting in meningoencephalitis, or isolated portions of them, as in tuberculoma, cerebral abscess, and encephalitis^(15)^. Tuberculous pachymeningitis can also occur, showing low signal intensity on T2-WI and diffuse contrast enhancement of the dura mater^(4)^, as depicted in Figure 3.

## IMMUNE-MEDIATED ETIOLOGY

Autoimmune encephalitis and limbic encephalitis are rare inflammatory conditions with similar clinical and radiological characteristics, distinguished by the specific subtype of neuronal antibody involved. They can be classified as paraneoplastic or non-paraneoplastic. In the case of an occult neoplasm, it is recommended that the patient be followed for up to four years after the diagnosis of autoimmune encephalitis.

**Figure 4:**
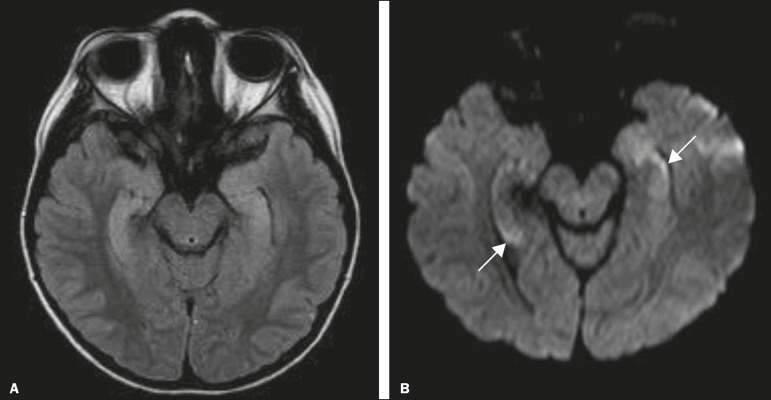
A 9-year-old female patient with behavioral changes, inversion of the sleep-wake cycle, and seizures. Tests for infectious and metabolic causes were negative. The cerebrospinal fluid tested positive for anti-GAD antibodies. Positron emission tomography/CT with 18F-fluorodeoxyglucose with no hypermetabolic changes suggestive of neoplastic involvement. Axial FLAIR-weighted MRI sequence and DWI (A and B, respectively) showing hyperintense signals in the mesial temporal regions with foci of restricted diffusion (arrows). A follow-up examination one year later showed a marked reduction in the volume of the hippocampal formations (image not shown).

Regardless of etiology, the involvement of the limbic system is the most characteristic finding of immune-mediated encephalitis, with T2/FLAIR hyperintensity in the cortical and subcortical regions of the temporal lobe, in most cases bilateral and asymmetric (Figure 4). In contrast to what is seen in herpes simplex encephalitis, immune-mediated encephalitis often involves the basal ganglia, whereas the lateral temporal lobe and the insula are generally spared. Restricted diffusion (on DWI) and hemorrhage are uncommon findings. Immune-mediated encephalitis can evolve to mesial temporal atrophy and temporal lobe epilepsy^(1,16)^.

## NEURODEGENERATIVE ETIOLOGY

**Figure 5:**
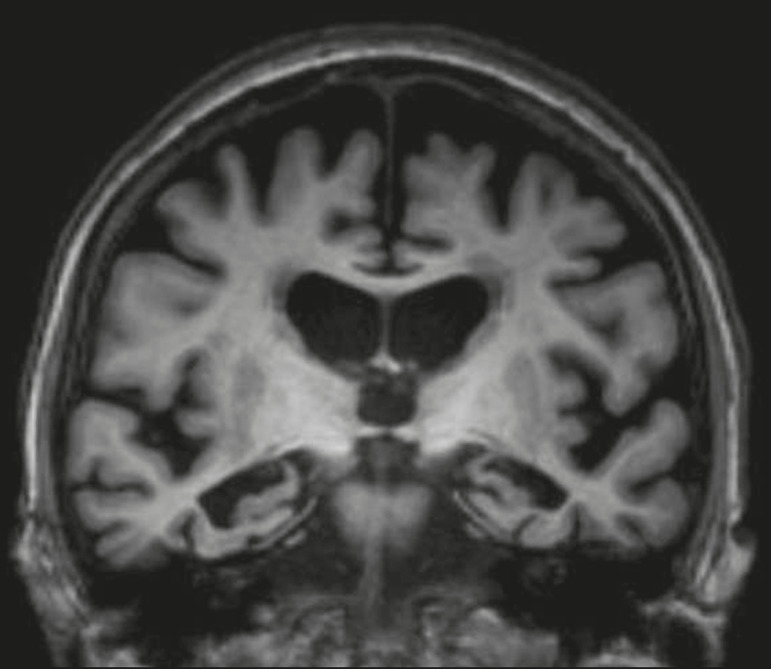
A 77-year-old female patient who presented with short-term memory loss and attention deficit. Coronal T1-weighted MRI sequence showing enlargement of the temporal horns and a reduction in the volume of the hippocampi.

Alzheimer's disease is the most common subtype of dementia, responsible for two thirds of all cases. The most common initial symptom is memory impairment, with or without executive and visuospatial dysfunction. Deposits of beta amyloid peptide and tau protein result in selective neuronal loss in the hippocampi and para-hippocampal gyri. As a result, there is a reduction in the volume of the mesial temporal lobe disproportional to the atrophy of the remaining cerebral parenchyma; this is the most characteristic finding in Alzheimer's disease (Figure 5). The mesial temporal lobe atrophy scale is a visual rating scale capable of quantifying the degree of hippocampal atrophy and has high sensitivity for the diagnosis of dementia due to Alzheimer's disease, although its specificity is low^(17)^.

**Figure 6:**
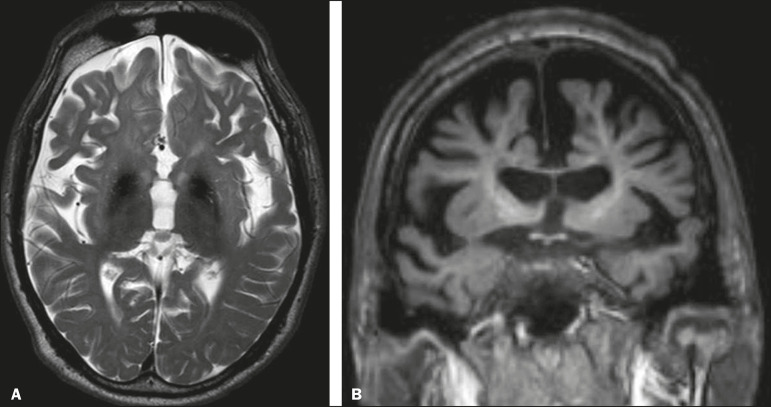
A 37-year-old male patient with progressive neurological deterioration. Axial T2-weighted and coronal T1-weighted MRI sequences (A and B, respectively) showing bilateral reduction in the volume of the encephalon beyond what would be expected for the age bracket, predominantly in the frontotemporal region, and preservation of the posterior cortical regions (A).

Frontotemporal dementia is one of the most common causes of pre-senile dementia. It is characterized by behavioral, personality, and language disorders, with selective degeneration of the frontal or temporal lobes. The two main variants are behavioral disorders and progressive primary aphasia. In the semantic variant of progressive primary aphasia^(1)^, the most common presentation is asymmetric atrophy of the left temporal lobe, as well as of the posterior portion of the frontal lobe and insula on the left (Figure 6).

## EPILEPSY SYNDROME

**Figure 7:**
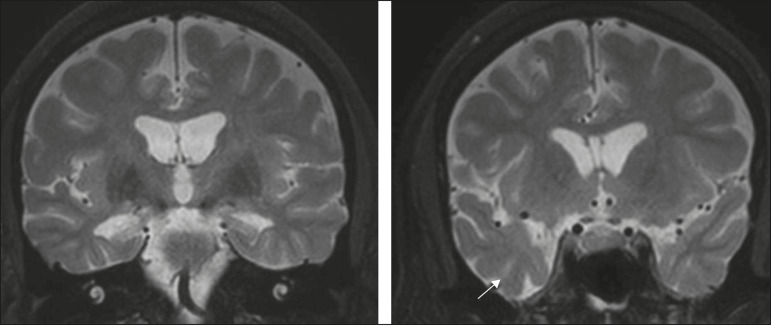
A 62-year-old male patient with refractory seizures. Coronal T2-weighted MRI sequence showing a reduction in the volume of both hippocampi, with a hyperintense signal and a loss of rugosity on the upper surface (cytoarchitectural alteration). Note the blurring of the transition between the gray and white matter in the right temporal pole (arrow).

Mesial temporal sclerosis is the most common etiology of temporal lobe epilepsy. Most patients present with difficult-to-control complex partial seizures. Temporal lobe epilepsy has also been associated with genetic factors, as well as with a history of febrile seizures, CNS infections, or limbic encephalitis. In temporal lobe epilepsy, there is neuronal loss and gliosis, predominantly involving the hippocampal regions. The findings on MRI include a loss of hippocampal volume with dilatation of the temporal horn and increased signal intensity on T2 and FLAIR-WI. Bilateral involvement, as depicted in Figure 7, is seen in 10% of cases. In up to 20% of the patients, there are accompanying lesions, including atrophy of the amygdala, fornix, mammillary body, and entorhinal cortex, as well as loss of grey-white matter interface in the anterior temporal lobe^(1,13,18)^.

## CEREBROVASCULAR ETIOLOGY

**Figure 8:**
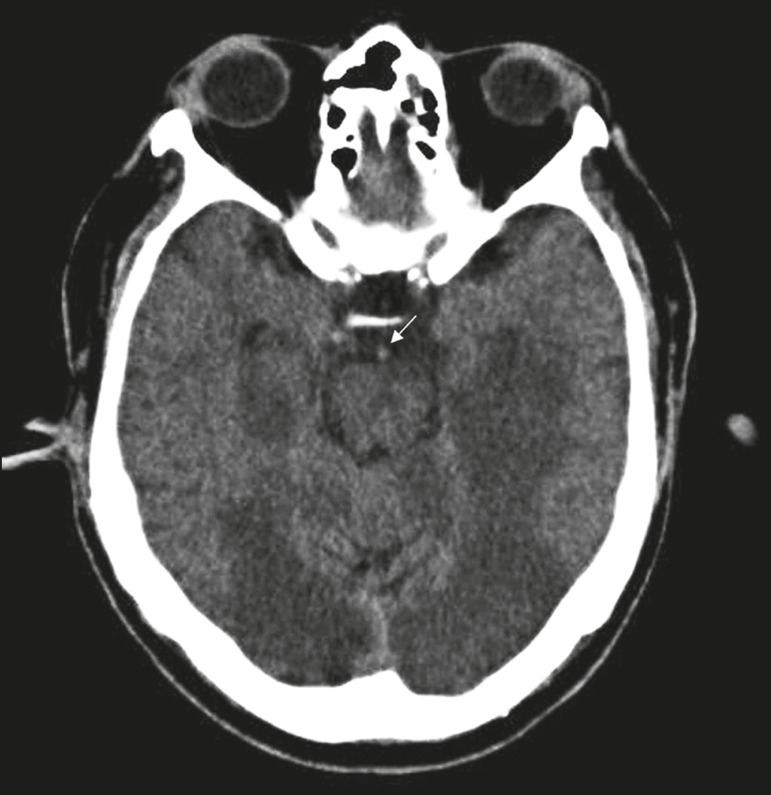
A 67-year-old male patient who presented with sudden-onset headache, blurred vision, and a reduced level of consciousness. Axial CT scan showing hypoattenuation of the cortical and subcortical regions, involving the medial temporal and occipital lobes, as well as the mesencephalon and cerebellum. Note the hyperdense artery sign, consistent with acute thrombosis, in the basilar artery (arrow).

The arterial supply of the temporal lobes depends as much on anterior circulation as on posterior circulation. Occlusion at the top of the basilar artery or in the posterior cerebral arteries can evolve to ischemia of the mesial temporal lobes, occipital lobes, mesencephalon, or thalamus. In acute cases, CT can reveal intravascular thrombosis (hyperdense artery sign), hypoattenuation (with a loss of differentiation between the cortical and subcortical components in the corresponding vascular territory), and cytotoxic edema with a locally expansile effect^(8,19)^, as shown in Figure 8. The temporality of the ischemic insult can be evaluated more precisely on MRI. On DWI, there is evidence of restricted diffusion in the very early stages. Exclusive involvement of the hippocampus is rare, and differential diagnoses such as status epilepticus should be considered.

## METABOLIC ETIOLOGY

**Figure 9:**
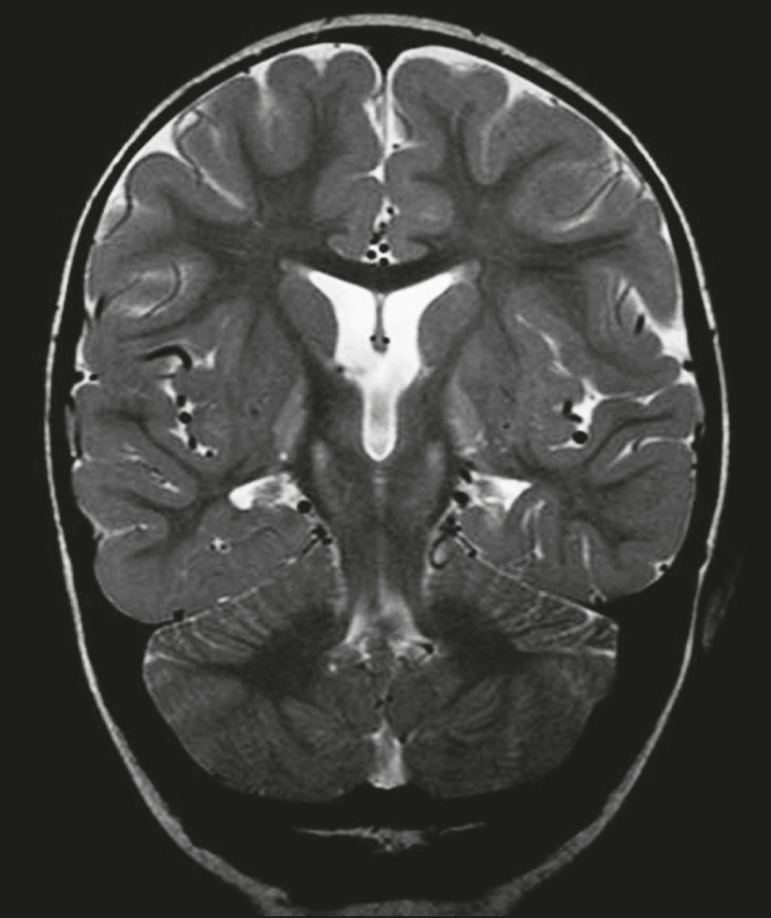
A 4-year-old female patient with a history of neonatal hyperbilirubinemia. Coronal T2-weighted MRI sequence showing a hyperintense signal in the globus pallidus, as well as, to a lesser extent, in the subthalamic, mesencephalic, and hippocampal regions (with reduced hippocampal volume).

Kernicterus is a rare condition in which there is neurological involvement secondary to hyperbilirubinemia (serum bilirubin > 20 mg/dL) with an accumulation of indirect bilirubin in the globus pallidus, subthalamic nuclei, hippocampus, putamen, thalamus, and cranial nerves (notably the third, fourth, and fifth cranial nerves). On MRI, findings include hyperintensity on T1-WI in the globus pallidus and subthalamic nuclei, progressing to T2/FLAIR hyperintensity in those structures, possibly leading to hippocampal atrophy^(20-22)^, as depicted in Figure 9.

## LEUKOENCEPHALOPATHY

**Figure 10:**
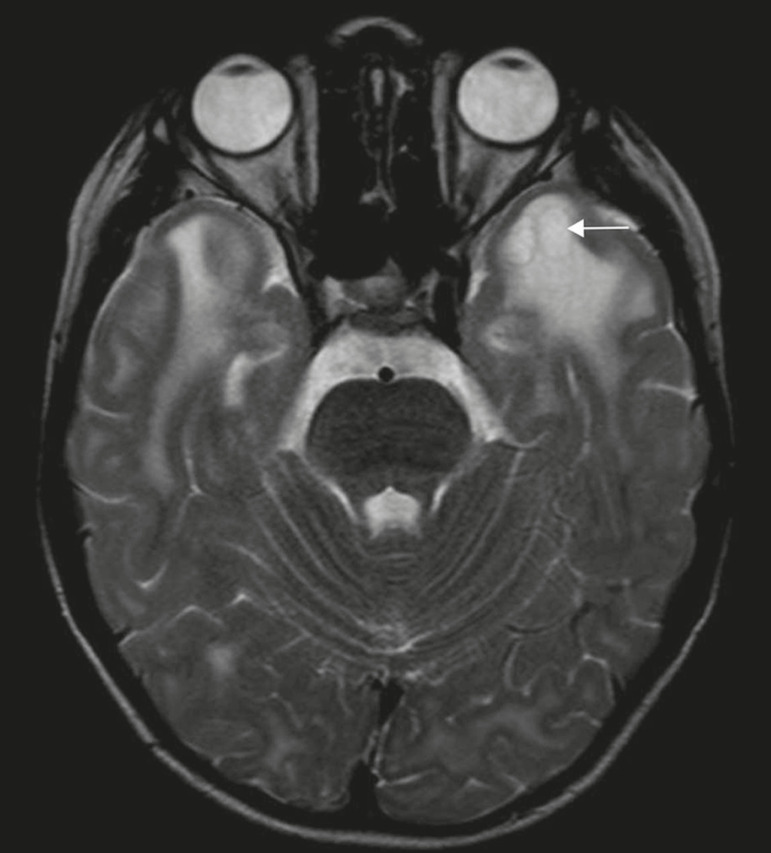
A 9-year-old male patient with macrocrania (since 5 months of age) and delayed neuropsychomotor development. Axial T2-weighted MRI sequence showing cysts in the left temporal pole (arrow). Note also the hyperintense signal in the subcortical and deep regions of the temporal lobes, as well as in the subcortical regions of the occipital lobe.

Megalencephalic leukoencephalopathy with subcortical cysts is an inherited autosomal recessive disease characterized by extensive vacuolization in the external layers of the myelin sheath. The clinical condition typically begins in infancy, presenting as macrocephaly accompanied by slow, progressive deterioration of motor function. The diagnosis can be established on the basis of clinical and imaging findings typical of the disease (Figure 10). On MRI, the affected white matter shows a diffuse, confluent hyperintense signal, although the basal ganglia and the cerebellar white matter have a normal aspect. Subcortical cysts initially appear in the temporal lobes, later being seen in the frontal and parietal lobes^(13,23)^.

## NEOPLASTIC ETIOLOGY

**Figure 11:**
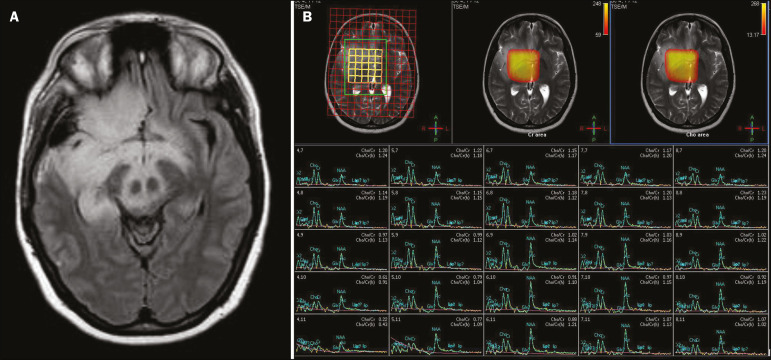
A 31-year-old female patient with a history of headaches and seizures. A: Axial FLAIR-weighted MRI showing a hyperintense signal in both temporal lobes and in the frontal lobes (more pronounced on the right), together with involvement of the mesencephalic parenchyma. In contrast-enhanced sequences, no enhancement was identified, nor was there any increased perfusion (images not shown). B: Multivoxel spectroscopy with an echo time of 144 ms, showing a choline/creatine ratio ≤ 1.23 and a trend toward a reduction in N-acetylaspartate. The patient underwent a temporal lobectomy and a right amygdalohippocampectomy, and the histopathology study showed a low-grade (grade II) diffuse fibrillary astrocytoma.

**Figure 12:**
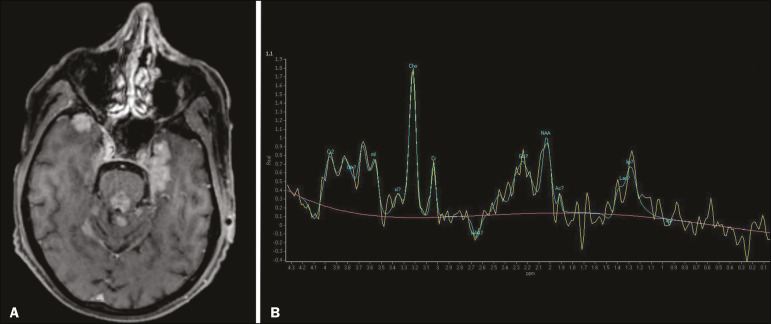
A 47-year-old male patient, diagnosed with high-grade B-cell non-Hodgkin lymphoma, who presented with sudden-onset diplopia, ataxia, and loss of balance. An initial analysis of the cerebrospinal fluid showed no relevant changes. A: Axial contrast-enhanced T1-weighted MRI sequence showing lesions with diffuse, intense enhancement in the anterior aspect of the temporal lobes, hippocampi, mesencephalic tegmentum and in the cerebellum. B: Point resolved spectroscopy with a short echo time (31 ms), showing a significant lipid/lactate peak in the lesions, with a solid aspect and homogeneous enhancement (which can be due to microscopic necrosis, suggestive of lymphoma, given that gliomas and metastases usually present such a peak in areas of low contrast enhancement). There was also an increase in choline levels, suggesting increased membrane turnover.

Neoplasms can involve the temporal lobes simultaneously, most commonly by dissemination via the anterior commissure, although also via the corpus callosum and the hippocampal commissure. Although this pattern is more common in glial tumors (Figure 11), it can occur in other contexts, such as in cases of secondary lymphoma (Figure 12). Low-grade diffuse astrocytomas (grade II) are slow-growing and are commonly supratentorial, typically affecting the frontal and temporal lobes. On CT, they appear as hypodense lesions without contrast enhancement. On MRI, they appear as lesions with a hypointense signal on T1 WI and a hyperintense signal on T2 WI, with a moderate expansile effect and no contrast enhancement. Low-grade diffuse astrocytomas (grade II) show no restricted diffusion on DWI, and their relative cerebral blood volume is fairly low (typically ≤ 1.75 times that of the contralateral parenchyma) on perfusion MRI^(1,23)^.

There is evidence that cerebral blood volume is lower in lymphomas than in glial neoplasms^(24)^. In most cases, lymphomas have high cellularity (with an isointense or hypointense signal on T2-WI), restricted diffusion on DWI, and intense contrast enhancement^(12,25)^.

## CONCLUSION

Various disorders can affect the temporal lobes bilaterally. Knowledge of those disorders and of their main aspects of imaging, with an emphasis on MRI, facilitates their early diagnosis, thereby potentially improving the prognosis.
